# Unsupervised Machine Learning for Advanced Tolerance Monitoring of Wire Electrical Discharge Machining of Disc Turbine Fir-Tree Slots

**DOI:** 10.3390/s18103359

**Published:** 2018-10-08

**Authors:** Jun Wang, Jose A. Sanchez, Izaro Ayesta, Jon A. Iturrioz

**Affiliations:** 1 College of Mechanical Engineering, Tianjin University of Science and Technology (TUST), Tianjin 300222, China; jwang003@ikasle.ehu.eus; 2 Aeronautics Advanced Manufacturing Center (CFAA), University of the Basque Country (UPV/EHU), 48170 Zamudio, Spain; izaro.ayesta@ehu.eus (I.A.); jonander.iturrioz@ehu.eus (J.A.I.)

**Keywords:** machine learning, tolerance monitoring, wire electrical discharge machining, aerospace, turbine manufacturing, fir‐tree slots

## Abstract

Manufacturing more efficient low pressure turbines has become a topic of primary importance for aerospace companies. Specifically, wire electrical discharge machining of disc turbine fir-tree slots has attracted increasing interest in recent years. However, important issues must be still addressed for optimum application of the WEDM process for fir-tree slot production. The current work presents a novel approach for tolerance monitoring based on unsupervised machine learning methods using distribution of ionization time as a variable. The need for time-consuming experiments to set-up threshold values of the monitoring signal is avoided by using K-means and hierarchical clustering. The developments have been tested in the WEDM of a generic fir-tree slot under industrial conditions. Results show that 100% of the zones classified into Clusters 1 and 2 are related to short-circuit situations. Further, 100% of the zones classified in Clusters 3 and 5 lie within the tolerance band of ±15 μm. Finally, the 9 regions classified in Cluster 4 correspond to situations in which the wire is moving too far away from the part surface. These results are strongly in accord with tolerance distribution as measured by a coordinate measuring machine.

## 1. Introduction

The manufacture of aircraft engines has increased dramatically in recent years. Some figures show that the number of passengers transported by air has seen continuous growth during the last decade, with some forecasts even predicting an exponential increase for the period 2020–2030 [1]. At the same time, the Advisory Council for Aviation Research and innovation in Europe (ACARE) have set a number of objectives including a 75% reduction in harmful COx and NOx emissions and a 65% decrease in noise levels by the year 2050 [2]. In order to comply with these strict requirements, special attention must be paid to aircraft engine components, amongst which the low pressure turbine (LPT) plays a critical role.

Manufacturing more efficient LPTs has become a topic of primary importance for aerospace companies. The efficiency in manufacturing an LPT is closely related to the manufacturing of its components. In fact, the number of high-added value parts in the turbine is very high. These components are characterized by their ability to withstand extremely high temperatures and pressures during their service life. Advanced materials such as nickel-based alloys and other super-alloys [3] are used to manufacture these parts. Several authors report on the low machinability of these alloys [3,4]. Moreover, very complex geometries, extremely good surface finish, and very tight tolerances are very common specifications in components such as the nozzle guide vane (NGV), the so-called blisks (blade integrated disks), or the disc turbine (fir-tree geometry slots). All of these facts explain why manufacturing, and specifically, machining of these advanced components is currently a topic of interest for both academia and industry.

**Figure 1:**
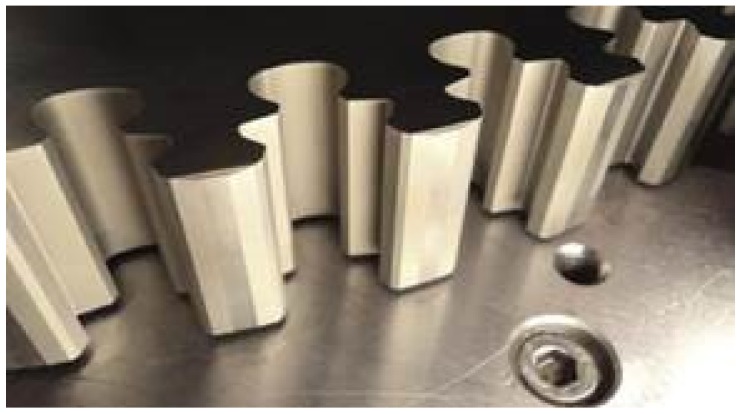
Example of geometry of a fir-tree slot.

The term fir-tree slot refers to the shape of the slot that must be machined on the turbine disc. The component and the manufacturing methods have been described and studied in detail by Klocke et al. [5]. In order to withstand the extremely high temperatures of the engine, low-machinability Nickel-based super-alloys such as Inconel 718 are used for manufacturing the turbine disc. Figure 1 displays the typical geometry of a fir-tree slot. 

The fir-tree slot exhibits low fillet radii, extremely tight tolerances (usually in the range ±5 μm or even less for some extreme applications) and controlled surface roughness below Ra1μm. Moreover, in many cases the slot shows tapering with respect to the plane of the disc. All these characteristics ensure a perfect match with the root of the turbine blade, thus enabling retention of the blade against the centrifugal forces acting when the disc is rotating at very high peripheral speeds. Taking into account that an aircraft engine may require many stages of discs (in excess of 40 in many instances), and that in some cases the number of blades can be as high as 200, it is unsurprising that so much attention is currently being paid to the manufacturing of this feature, since it accounts for a significant part of the cost of the engine. 

Broaching of fir-tree slots on turbine discs is currently accepted as an optimum machining process [5]. It is applied in industry mainly due to the high machining speed, but also due to the excellent surface properties, which have a critical influence on the fatigue life of the component. This is due to the compressive residual stresses and the controlled surface roughness that the broaching process generates on the component. Moreover, accuracy requirements are optimally met. However, limitations of the process include high tooling and machinery costs, high set-up times, increased wear of broaching tools when machining Inconel 718, and the low flexibility of the process [6]. 

At this point, the wire electrical discharge machining (WEDM) process has emerged as a promising alternative to broaching. In the WEDM process, a brass copper wire is fed between two guides in the presence of a dielectric medium, normally deionized water [7]. The wire follows a programmed NC path while generating electrical discharges that remove material from the work-piece, which must be electrically conductive. The process is particularly well-suited to the machining of extremely hard materials, or those of low machinability (such as hardened steels, super-alloys, conductive ceramics), in which complex geometries, tight tolerances, and excellent surface finish can be obtained by carrying out successive trim cuts.

Jet engine manufacturers now regard WEDM as the process that can overcome some of the limitations shown by broaching for the production of fir-tree slots. For instance, the process is economical, flexible, and can be easily automated. Although in the past, due to its thermal nature, WEDM has been disregarded for the manufacturing of critical engine components, many recent studies [5,8,9,10,11,12] and industrial applications [13] have shown that the latest machines equipped with low-energy generators can provide an alternative to broaching in terms of expected service life of the component. 

However, important issues must be still addressed for optimum application of the WEDM process for fir-tree slot production. Strict tolerance bands throughout the entire profile, the absence of wire marks on the surface of the component, and manufacturing traceability (imposed by aerospace industry) are state-of-the-art research topics. In this regard, the work carried out by Klocke et al. [14] is particularly relevant. These authors developed a novel monitoring system for the second trim cut, according to the fact that this is carried out in iso-frequency mode and therefore gap control is not possible using the servo reference voltage. The authors established threshold limits for the mean voltage that ensure the finish and integrity of the specified surface, so that damage on the machined surface could be detected on-line. Experiments are required prior to operation to identify the threshold values for each specific application. 

The development of machine learning (ML) techniques in recent years has opened up a new perspective on the problems of process monitoring and traceability through the use of large quantities of data incoming from the process. ML and AI techniques have been successfully applied to the modeling of the manufacturing process, mainly due to their ability to deal with complex multivariable and non-linear phenomena [15]. Examples of interesting research works can be found in the literature, dealing, for instance, with the comparison between analytical models and ANN techniques for modeling turning operations [16]. ANNs have been used by Caggiano et al. [17] to obtain a deeper insight into the phenomena involved in the WEDM process. These authors proposed using artificial neural networks (and more specifically, a multi layer perceptron network) for regression in order to monitor the WEDM process. Zhen et al. [18] developed a spark monitoring system for pulse identification in which discharge features are extracted using support vector machine (SVM) and random forest (RF) techniques. Recent research works in the field of WEDM have also been based on supervised techniques, such as ANN [19] and SVM [20], but to the best knowledge of the authors, unsupervised ML techniques have not been applied to the WEDM process yet. 

However, the application of unsupervised learning to manufacturing processes, and more specifically, to the manufacturing of turbine components is still scarce. When using unsupervised learning techniques, labeling can be avoided and the amount of data for training can be largely reduced. Undoubtedly, the development of unsupervised ML techniques for clustering to engineering problems shows high potential. In production operations it is difficult to obtain a sufficiently large number of labeled data (thousands of costly experiments would be required, for instance, to use deep learning techniques), which opens up the possibility of successfully applying unsupervised learning techniques to industrial applications.

Some examples of unsupervised learning in industrial data can be found in the field of supply chains and production logistics [21]. The K-means technique has been successfully applied to increase the output in semiconductor production, as shown in Reference [22]. Further, K-mean variation has formed the basis of research aimed at improving refinery catalytic processes [23]. A very recent study [24] proposed the use of a cyber-physical system that uses actual data from a machining process. The objective is to evaluate the performance of a machine tool spindle during a high-performance machining operation. In this study three clustering methods were examined, namely K-means, hierarchical agglomerative, and Gaussian mixture. 

Clustering is an important task within unsupervised learning [25]. A considerable number of techniques have been proposed in the literature for clustering, amongst which the already mentioned K-means [26] and hierarchical clustering [27,28] are probably the most widely used. One characteristic of K-means is that this algorithm is simple and easy to understand. Similarly, the operation speed is fast, but it can only be applied to continuous data. As a limitation, the number of the groups needs to be assumed before clustering, which is not always easy although this can also be solved by using various metrics [29]. Unlike K-means, hierarchical agglomerative clustering [30] does not require the assumption of a fixed number of groups prior to clustering. Furthermore, hierarchical clustering has the advantage of prompting the entire clustering process to form a clustering tree at once. Once the tree is formed, the number of clusters can be directly decided by the user as a function of required accuracy. If the number of clusters changes, calculating the attribution of data points is not needed again. Since the distances between all the multiple clusters have to be calculated, the computational complexity of hierarchical clustering could be considerable in comparison with other methods. 

In this work, a novel approach is proposed for monitoring the production of disc turbine fir-tree slots by WEDM. The approach is based on the use of unsupervised machine learning techniques. The main contribution of this work with respect to previous works is to explore the possibility of automatically monitoring component tolerances, which are very strict in fir-tree slot production. The ability of these ML techniques to classify the different regions of the fir-tree slot geometry as a function of the achieved tolerances eliminates the need for preliminary time-consuming experiments to set up threshold limits. Basic experiments reveal that unsupervised clustering techniques—namely K-means and Hierarchical Clustering—are very efficient in producing clusters as a function of the distance between wire and work-piece, or in other words, as a function of the final deviation of wire path and nominal profile. As a result, the approach becomes general and can be easily automated in machine control.

Section 2 presents the materials and methods for the research, setting the procedure for the fundamental experiments of correlation between process signals and wire infeed for the monitoring approach. Section 3 collects the results from the fundamental experiments, from which valuable conclusions can be obtained about the theoretical capability of the proposed monitoring techniques. Discussion of results is presented in Section 4. Both hierarchical clustering on distribution curves of ionization time, and K-means on features extracted from those curves, exhibit sound correlation with wire infeed during the fundamental experiments. Finally, the industrial feasibility of the proposal is presented in a generic fir-tree geometry cut under industrial conditions. Clusters predicted by hierarchical clustering are compared in 30 different zones of the profile with part tolerances as measured using a coordinate measuring machine. The use of ionization time-based unsupervised ML is shown to be a very effective way of classifying the various lobes and linear parts of the fir-tree depending on the actual distance between wire and part during the second trim cut, and therefore, on the final part tolerance.

## 2. Materials and Methods

Since the second trim cut does not modify the geometry of the part, local damage on the fir-tree profile previously caused by the roughing and/or the first trim cut will result in modifications of the gap during the second trim cut. If, for instance, during the roughing cut, excessive wire-lag removes too much of the part material at a convex lobe of the fir-tree, during the second trim cut the distance between wire and machined surface at that exact point of wire path will be higher than ideally expected. Most high-quality WEDM machines are equipped with an iso-frequency pulse generator for the second trim cut, which maintains constant both the off-time and pulse time of each pulse. Since the feed rate is constant and is established by machine table look-up, the generator cannot control gap dimension. As a consequence, if an excess or lack of material is present (due, for instance, to a problem during the roughing cut), instabilities, marks, and deleterious surface finish may occur at that point, thus affecting the quality of the part. Taking into account that infeed is a measure of the actual distance between wire and machined surface, it can also be used to define the final tolerance of the profile. This is due to the fact that the second trim cut does not modify the geometry, which is instead dependent on the quality of the roughing and the first trim cut.

Fundamental experiments have been carried out on an ONA AV-35 WEDM machine (CuZn37 0.25 mm diameter brass wire), which is the industrial equipment for disc turbine production. Part material is Inconel 718.

Electrical parameters for the second trim cut were selected by machine table look-up, and are listed in Table 1. Infeed values for the experiments are modified in 5 different regions of the sample part (see Table 2), ranging from −8 μm to 32 μm, with a variation of 10 μm between every two consecutive regions of the sample.

For every region of the experiment, the voltage signals were directly measured from the WEDM machine generator by using a Tektronix ThDP0200 Differential Probe. Data were collected using a Tektronix 5034B Digital Oscilloscope, which allows for a very high sampling rate of 50 MS/s. Signal resolution is therefore 20 ns, which is sufficiently high to detect process-related events that may appear in the voltage signal. 30 trains of voltage pulses of 0.1 ms were recorded in each region directly from the WEDM process under actual industrial conditions. 

**Figure 2:**
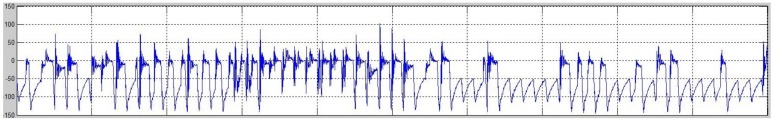
Voltage signal (time on horizontal axis) as registered by the oscilloscope during the second trim cut.

High frequency voltage signals have been collected as explained above, and thus the hypothesis of a possible correlation between voltage and wire infeed can be addressed. Figure 2 shows an example of a raw voltage signal, as registered by the oscilloscope. No signal filtering was applied to the recorded data.

## 3. Results

**Figure 3:**
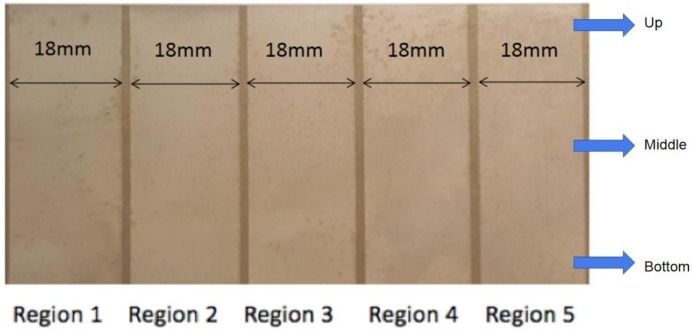
Surface appearance of the different regions as a function of wire infeed.

As a result of the experimental tests, surface finish was measured at three heights of each region, thus allowing for detecting the deformation of the wire related to excessive distance to the machined surface. The potential occurrence of short-circuits due to an excess of part material also becomes apparent from surface finish measurements. Figure 3 and Table 2 show the results of the experiment.

**Figure 4:**
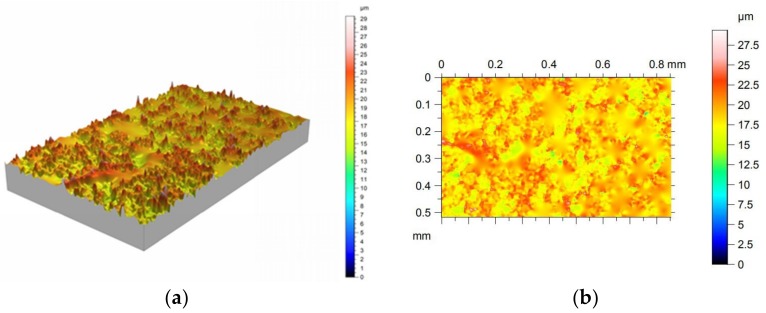
(**a**) 3D Surface topography of Region 3 as measured using the optical profilometer; (**b**) top view of the measured surface.

Surface roughness has been measured using a Leica optical roughness measurement instrument. Figure 4a shows the 3D surface topography corresponding to Region 3 (see Figure 3), whereas Figure 4b displays the top view of the measured surface. From the 3D description of the surface, statistical parameters such as Ra can be obtained. The complete roughness results corresponding to the different regions are collected in Table 2.

The voltage signal shows patterns that help to understand the appearance of the different regions of the machined surface. Region 1 is characterized by a relatively large distance between wire and work-piece. As is well-known, the wire loses straightness and cleaning of the gap becomes difficult in the upper and bottom zones. This explains the variation in surface finish for a given set of cutting conditions. In this case, failure discharges occur, in which although ionization has started, no current flow finally occurs during the on time. Regions 2, 3, and 4 exhibit a more uniform pattern. Effective discharges occur, and straightness of the wire is ensured. Finally, in Region 5 the wire is too close to the machined surface, producing short-circuits that increase surface finish.

Inspection of the characteristics of the voltage signal (see Figure 2), recorded during the experiments, reveals that ionization time *T_d_* plays a fundamental role in the second trim cut. Trains of discharges of “large” ionization time can be observed, which are related to an excessive distance between wire and work-piece. Further, short-circuit situations occur during which contact between wire and machined surface show instantaneous ionization. Therefore in the current work, it is proposed to use the distribution of ionization time (which can be directly obtained from the voltage signal) as the main indicator. Since the erosion mode is iso-frequency, it becomes clear that the largest possible ionization time is equal to pulse time, whilst the shortest possible ionization time corresponds to short-circuit situations. Between these two limits, a distribution of discharges as a function of ionization time can be obtained. 

**Figure 5:**
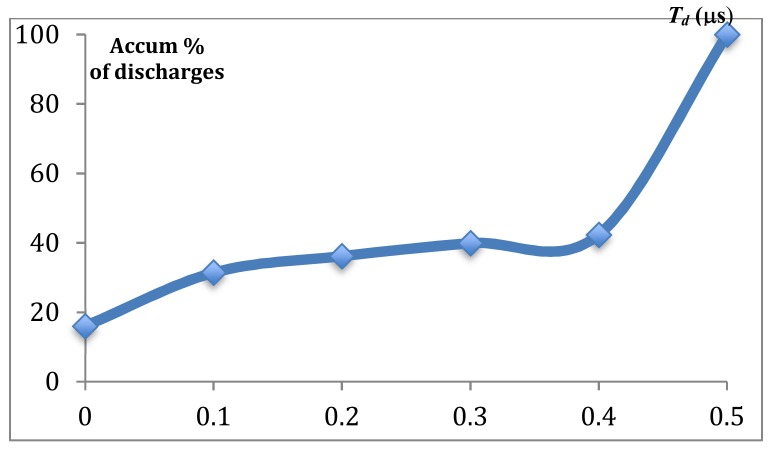
Abbot-Firestone curve of accumulated occurrence of types of discharge as a function of *T_d_* (μs).

From the oscilloscope measurements, values of *T_d_* for each single discharge can be extracted. As a result, the curve of distribution of *T_d_* (curve of Abbot-Firestone, see Figure 5) can be easily obtained, which represents the accumulated % of discharges (vertical axis) as a function of *T_d_*. The origin of the horizontal axis corresponds to *T_d_*= 0 (short-circuit situations), and as *T_d_* increases, the possibility of a failure discharge also increases. In this example, which corresponds to Region 1, it can be observed that approximately 42% of the discharges exhibit values of *T_d_* smaller than 0.4 μs. In other words, around 60% of discharges correspond to situations during which the wire is too far from the work-piece surface, resulting in failure discharge.

Therefore, by following this approach, curves for the different regions have been obtained. In the following paragraphs, the feasibility of using this new indicator for tolerance monitoring using unsupervised ML techniques will be addressed.

## 4. Discussion

**Figure 6:**
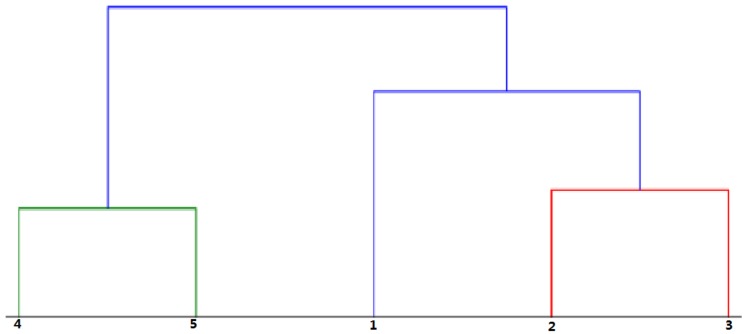
Result of HC applied on the curves of *T_d_* distribution for the different regions of the experiment.

Hierarchical clustering (HC) can be applied on the curves defined in the previous section and obtained from the fundamental experiments. Python 3.6 and Pycharm community are adopted as programming language and software respectively for HC. This technique forms a clustering tree at once, so that that the number of clusters is not fixed a priori. The results of HC on complete voltage sequences are listed in Table 3. Figure 6 shows the graphical plot, which aims to show the distance between the different clusters.

Two large clusters have been created. Group 1 includes Regions 4 and 5, characterized by large values of wire infeed (higher than 20 μm). Clearly separated from Group 1, Group 2 includes Regions 1, 2, and 3, in which wire infeed ranges from −8 μm to 12 μm. When compared with Table 3, there is a clear improvement derived from using *T_d_* distribution to detect variations of wire infeed. 

In order to confirm this result, the K-means technique has also been applied using features extracted from *T_d_* distribution. The objective is to finally decide the most suitable technique for tolerance monitoring not only in basic experiments, but also in industrial case studies. The selection of the optimum features was carried out by using the Pearson correlation coefficient (PCC), which is a well-known measure of the linear correlation between two variables, and it takes values from +1 to −1. The following features were selected for the analysis from the *T_d_* distribution: average (*Avg*), standard deviation (*Std*), skewness (*Skw*), and kurtosis (*Krt*). Table 4 displays the results, showing that in this case, the average and the skewness are the most relevant features. It can be noted that PCC values are very close to 1 (−0.999 for average, 0.915 for skeweness) than those presented in Table 4 (−0.479 for kurtosis, 0.495 for skeweness), which reveals a high degree of correlation between *T_d_* and wire infeed. 

Thus, using the values of average and skewness, K-means simulations, were run with different numbers of clusters. Numbers of pre-assumed clusters from 2 to 9 have been tried, with the highest scores corresponding to 2 clusters (0.6644), 3 clusters (0.4657), and 4 clusters (0.4546). Clustering has therefore been carried out assuming 2 and 3 clusters. The results are presented in Table 5. It is evident that 2 clusters are not sufficient to adequately separate the different regions. Good results are obtained when using 3 clusters. In this case, Region 1 (wire infeed −8 μm) has been included in a separate cluster. Regions 2 and 3 (wire infeed from 2 μm to 12 μm) have been classified in the second group. Finally, the regions characterized by the highest values of wire infeed (regions 4 and 5, 22 μm and 32 μm respectively) have been included in the third group. 

**Figure 7:**
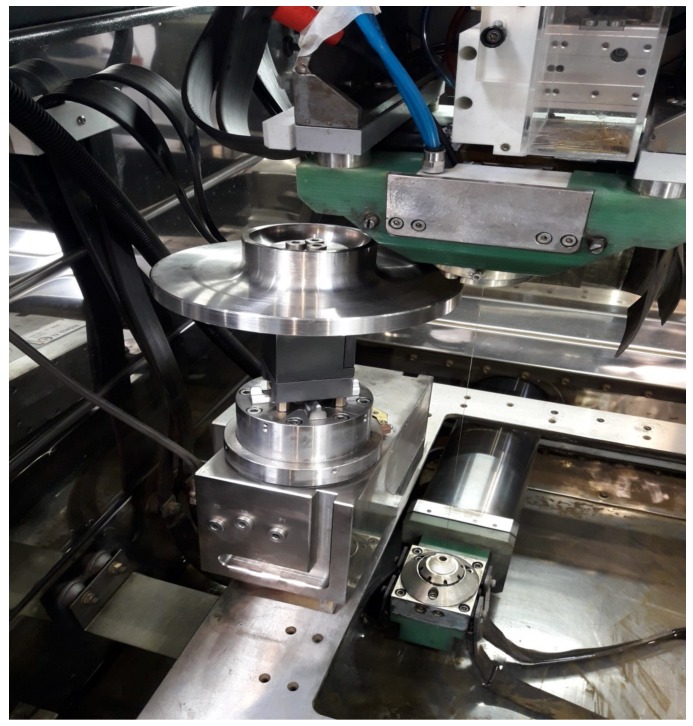
Set-up of the Inconel 718 prototype of the disc turbine mounted on the machine before WEDM’ing.

From these preliminary conclusions, the practical validation of the proposed methodology for tolerance monitoring on a generic fir-tree slot geometry subjected to WEDM under actual industrial conditions can be addressed. A photograph of the set-up of the Inconel 718 prototype of the disc turbine mounted on the machine before WEDM, is presented in Figure 7.

**Figure 8:**
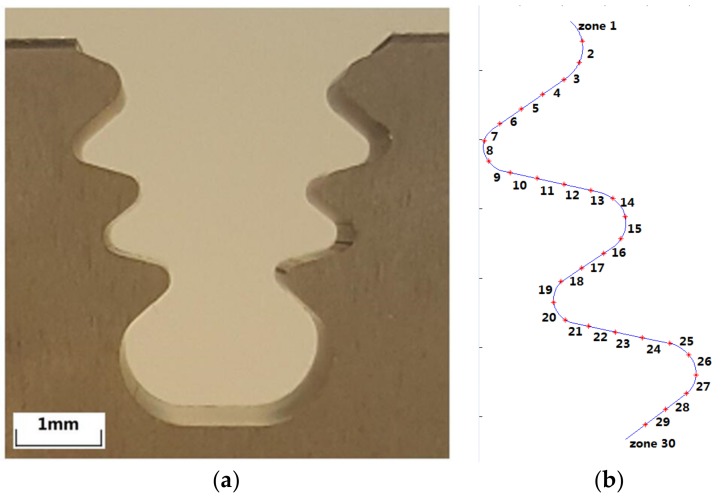
(**a**) Geometry of the generic fir-tree slot for industrial tests; (**b**) The profile is divided into 30 zones in which CMM measurements are compared with clustering results.

Figure 8 shows the geometry of the fir-tree slot selected for the industrial tests. On the right hand side (Figure 8a), a photograph of the component is shown. Figure 8b represents the control zones selected on the profile. At those zones the results from the novel monitoring technique will be compared with actual the tolerance measurements as obtained using a coordinate measuring machine (CMM), which is the current industrial measuring equipment for this kind of component.

As explained above, in order to test the accuracy of the clustering technique, tolerance deviations on the machined fir-tree slot geometry were also measured using a Mitutoyo 9106 coordinate measuring machine. A tolerance band of ±15 μm has been represented on the nominal profile obtained from the CAD file, so that the geometry of the deviations can be easily understood. Part thickness is 15 mm, and WEDM electrical parameters are selected by machine table look-up (similar to those used in the preliminary experiments, see Table 1), involving the first roughing cut and 2 trim cuts. For the tests, Inconel 718 was used as part material.

Although during the preliminary experiments both unsupervised ML techniques (K-means and HC) have shown efficiency and sufficient sensitivity to detect variations in wire infeed (and thus final part tolerance after the second trim cut), it was finally decided to use hierarchical clustering for simplicity. Since there is no need for feature extraction, and data regarding the *T_d_* distribution can be directly obtained from the voltage signal, HC is expected to provide a simple and at the same time effective method to classify the different regions of the fir-tree as a function of the final tolerance. Consequently, *T_d_* distribution is obtained at each of the 30 zones in which the profile has been divided and HC is applied on the collected data. The results are presented in Table 6.

Even though the geometry of the error throughout the profile is variable and cannot be characterized only by the maximum value, the efficiency of the technique can be quantified and interesting conclusions can be drawn from Table 6. Using 5 clusters allows for a better representation of the different zones in which the fir-tree geometry has been divided, and 100% of the zones classified into Clusters 1 and 2 are related to short-circuit situations. The maximum error is positive in all the cases, being always clearly higher (above 20 μm) than the tolerance band (+15 μm). Furthermore, 100% of the zones classified in Clusters 3 and 5 lie within the tolerance band of ±15 μm. Finally, the 9 regions classified in Cluster 4 correspond to situations in which the wire is moving too far away from the part surface. In other words, the error is negative in all of the cases, from which 44% are outside the tolerance band. In all the cases the error is more negative (−9 μm) than that corresponding to the regions classified in Clusters 3 and 5 (−8 μm).

**Figure 9:**
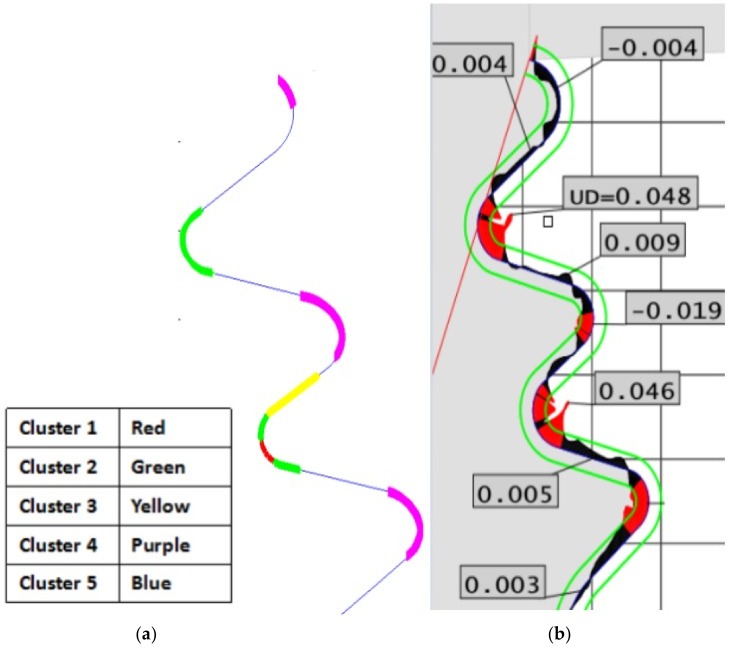
(**a**) Graphical representation of the clusters provided by hierarchical clustering; (**b**) Geometry of the error throughout the fir-tree profile, as measured by the CMM.

Figure 9 represents the clusters proposed by the HC method (Figure 9a) and the geometry of the error as measured with the CMM (Figure 9b). An excellent agreement between the predictions of the HC method and the geometry of the error can be found. For the sake of simplicity, different colors have been assigned to the 5 Clusters in which the HC solution has been presented. As shown in the figure, the zones corresponding to the concave lobes have been merged into one single cluster (purple color). These regions are characterized by the fact that the roughing and the first trim cut remove more part material than required, and therefore, during the second trim cut the gap between wire and machined work-piece is larger than ideally expected. The zones corresponding to convex lobes have been included in Clusters 1 and 2 (green and red). In these cases, short-circuit situations appear due to the fact that the wire “finds” a greater quantity of material than that ideally expected. Finally, linear zones have been classified into Clusters 3 and 5, where the process is within the tolerance band.

## 5. Conclusions

Wire electrical discharge machining of fir-tree slots for aerospace applications is currently a hot topic of research. Tolerances in this type of components are within the strongest requirements imposed on the machining processes. A novel approach for tolerance monitoring using unsupervised machine learning techniques has been presented. In order to avoid time-consuming experiments for establishing threshold values for the monitoring variable, the use of unsupervised machine learning techniques (namely K-means and hierarchical clustering) were examined in this study. The possibility of using distribution ionization time was studied through preliminary experiments. Hierarchical clustering of ionization time distribution curves appears to be efficient at classifying the regions as a function of wire infeed. Additional features were extracted from the curves of ionization time. Results from the Pearson correlation coefficient show the highest values for average and kurtosis. Classification of the regions using K-means with these features shows very good agreement with wire infeed. The proposed technique was validated by applying the process of WEDM to an actual fir-tree slot geometry under industrial conditions. The results from the clustering technique (hierarchical clustering) were compared with actual deviations as measured using a CMM. Using 5 clusters allows for a better representation of the various zones in which the fir-tree geometry was divided. It was found that 100% of the zones classified into Clusters 1 and 2 are related to short-circuit situations. The maximum error is positive in all the cases and is always clearly higher (above +20 μm) than the tolerance band (+15 μm). Further, 100% of the zones classified into Clusters 3 and 5 lay within the tolerance band of ±15 μm. Finally, the 9 regions classified into Cluster 4 correspond to situations in which the wire is moving too far away from the part surface (error more negative than −9 μm in all cases).

## Figures and Tables

**Table 1:** Electrical parameters for the second trim cut (taken from ONA AV35 WEDM machine).

Parameter	Value
Servo (V)	10
Pulse time (μs)	80
Current (A)	4
Open circuit voltage (V)	90
Off-time (μs)	1

**Table 2:** Infeed values and surface finish for each region at different heights.

Region	Infeed	Surface Finish Ra (μm)		
	(μm)	Bottom	Middle	Up
1	−8	1.3	0.9	1.1
2	2	1.0	0.9	1.1
3	12	0.9	0.9	0.9
4	22	0.9	0.8	0.9
5	32	1.2	1.4	1.4

**Table 3:** Hierarchical clustering (HC) of the different regions using the curves of distribution of *T_d_* for classification.

Group	Region	Infeed (μm)
Group 1	4	22
	5	32
Group 2	1	−8
	2	2
	3	12

**Table 4:** Results from the correlation analysis (*PCC*) between wire infeed and features from *T_d_* distribution: average (*Avg*), standard deviation (*Std*), skewness (*Skw*) and kurtosis (*Krt*).

	*Avg*	*Std*	*Skw*	*Krt*	*Wire Infeed*
***Avg***	1	0.079	−0.995	−0.948	−0.999
***Std***	0.079	1	0.019	−0.382	−0.041
***Skw***	−0.995	0.019	1	0.915	0.998
***Krt***	−0.948	−0.382	0.915	1	0.937
***Wire Infeed***	−0.999	−0.041	0.998	0.937	1

**Table 5:** Results obtained from K-means assuming 2 clusters and 3 clusters.

Region	*Avg*	*Std*	*Wire Infeed* (*μm*)	2 Clusters	3 Clusters
**1**	0.491	−0.213	−8	0	0
**2**	0.445	0.192	2	0	1
**3**	0.394	0.524	12	0	1
**4**	0.336	0.823	22	1	2
**5**	0.283	1.13	32	1	2

**Table 6:** Hierarchical clustering using *T_d_* distribution of the 30 zones in which the fir-tree profile was divided for the analysis.

Zones	Maximum Error (μm)	3 Clusters	4 Clusters	5 Clusters
1	−9	3	3	4
2	−2	3	3	5
3	−8	3	3	5
4	−4	3	3	5
5	−4	3	3	5
6	−8	2	2	5
7	20	2	2	2
8	40	2	2	2
9	48	3	3	2
10	2	3	3	5
11	2	3	3	5
12	9	3	3	5
13	−10	3	3	4
14	−12	3	3	4
15	−19	3	3	4
16	2	3	4	5
17	6	3	4	3
18	6	2	2	3
19	20	1	1	2
20	46	2	2	1
21	46	3	3	2
22	9	3	3	5
23	8	3	3	5
24	5	3	3	5
25	−15	3	3	4
26	−18	3	3	4
27	−20	3	3	4
28	9	3	3	5
29	8	3	3	5
30	6	3	3	5
